# Association between Chronic Urticaria and *Helicobacter pylori* Infection among Patients Attending a Tertiary Hospital in Tanzania

**DOI:** 10.1155/2020/5932038

**Published:** 2020-09-01

**Authors:** Magdalena F. Dennis, Daudi R. Mavura, Luryritha Kini, Rune Philemon, Elisante J. Masenga

**Affiliations:** ^1^Department of Dermato-Venereology, Kilimanjaro Christian Medical University College, P.O. Box 2240, Moshi, Tanzania; ^2^Department of Paediatrics and Child Health, Kilimanjaro Christian Medical University College, P.O. Box 2240, Moshi, Tanzania

## Abstract

**Background:**

Chronic urticaria (CU) is a common skin disease; however, its etiology is rarely recognized. Infection due to *Helicobacter pylori* (*H*. *pylori*) has been shown in some studies to play a significant role in the pathogenesis of CU.

**Objective:**

This study was conducted to determine the association between CU and *H*. *pylori* infection among patients attending the Regional Dermatology Training Center, Northern Tanzania, from October 2018 to April 2019. *Methodology*. A matched case-control study that included 55 cases and 55 controls matched by age and sex was conducted. Data were collected through direct interviews, and the results of laboratory investigations were recorded in the extraction sheet. An enzyme-linked immunosorbent assay test was used to detect *H*. *pylori* antigen in the stool samples. Conditional logistic regression was used to measure the association between CU and *H*. *pylori*.

**Results:**

The total number of participants in this study was 110 patients (55 cases and 55 controls), whereby the median age was 31 (IQR 27–45) among controls versus 34 (IQR: 22–46) years among the cases. Both cases and controls had the same number of females and males. There was no significant association between CU and baseline characteristics of the participants. There was an association between CU and *H*. *pylori* infection, such that subjects with CU had a higher number of positive *H*. *pylori* test (15/55 = 27%) versus controls (6/55 = 10.1%) (*p* = 0.0225). The adjusted odds of CU among patients who were positive for *H*. *pylori* were sixfolds higher (OR = 6.9; CI: 1.3–36.2; *p* = 0.021) than those of patients who were negative for *H. pylori.*

**Conclusion:**

There was a strong and significant association between CU and *H*. *pylori* infection. We recommend investigating for *H*. *pylori* in all cases of CU and conducting further trials on *H*. *pylori* eradication.

## 1. Introduction

Urticaria, commonly known as hives, is defined by the appearance of short-lived swellings, which are called wheals. This condition presents with either wheals, angioedema, or both [1]. Patients with urticaria present with severe itching that can have effect on the quality of their life [2]. Urticaria can be either acute or chronic. Acute urticaria occurs days to weeks, producing wheals that rarely may last more than 12 hours, and complete resolution of the lesions may occur within six weeks of onset. In comparison, daily episodes lasting more than six weeks are designated as CU [3]. Both children and adults can develop urticaria, although it is more common in adults, and females are more affected than males [4]. Urticaria is a debilitating condition affecting 0.3% to 5% of the general population worldwide [5]. In sub-Saharan Africa, the prevalence of CU is estimated at 1.9% [6]. In Tanzania, there is a paucity of information on CU; however, urticaria accounts for 1.2% of skin diseases among pediatric patients [7]. CU may result from several causes; some are known as bacterial, viral, fungal, and protozoan agents; however, the etiology, for most cases, remains unknown [8]. In recent years, there has been emerging literature associating CU and *H*. *pylori* infection [4]. Several theories have been put forward for this association. For example, it is thought that infection with *H*. *pylori* increases the permeability of the stomach lining and thus increases the exposure to allergens in the gastrointestinal tract [3]. Also, the immune response to *H*. *pylori* produces antibodies that may encourage the release of histamine in the skin [9]. However, other authors suggest that this association is still controversial [4]. *H*. *pylori* is a Gram-negative bacterium that is found on the luminal surface of the gastric epithelium. This bacterium is transmitted through the fecal-oral route and has been associated with low-social economic status, poor hygiene, and consumption of untreated water [10]. This bacterium can cause gastric inflammation of the underlying mucosa leading to peptic ulcer, gastritis, and gastric mucosa-associated lymphoid tissue (MALT) lymphoma. Most of the infected people are asymptomatic, and they develop manifestations of peptic ulcer. Extra-gastrointestinal manifestations such as CU, vascular, and autoimmune disorders occur later in life [9]. CU has continued to be a frustrating condition to both the patient and the care provider and has a significant impact on the patient's well-being especially on sleep, mood, and work. CU hence impacts economically, leads to social isolation, degrades the quality of life, and is enormously challenging to the managing clinician [11]. Management of CU focuses mainly on nonpharmacological and pharmacological measures [12]. In Tanzania, there is a paucity of information on the association between CU and *H*. *pylori* infection, and there is no specific management of patients with CU. Patients are managed according to their signs and symptoms [13]. There is a need to determine the extent of association between CU and *H*. *pylori* infection in our setting for effective management of CU.

## 2. Materials and Methods

### 2.1. Study Area and Design

This study was a case-control,which was conducted at Kilimanjaro Christian Medical Centre (KCMC) in Moshi-Tanzania from October 2018 to April 2019. KCMC is a referral, consultant, research, and teaching hospital located in north-eastern Tanzania and lies at the foot of Mount Kilimanjaro. RDTC is one of the departments at KCMC which offer advanced diploma and specialist degree training in Dermatovenereology in East and Central Africa.

### 2.2. Study Population

#### 2.2.1. Case Definition

Individuals aged five years and above who attended RDTC and were clinically diagnosed with CU were selected during the study period. Cases were recruited from the outpatient department at RDTC. CU was diagnosed as a patient presenting with the following features: recurrent, transitory, and itchy wheals that occur daily or almost every day and persist for longer than six weeks.

#### 2.2.2. Control Definition

Individuals aged five years and above who attended RDCT and were clinically diagnosed with other dermatological conditions and free from any feature of urticaria were selected. Controls were recruited from the outpatient department at RDTC.

#### 2.2.3. Matching

Cases and controls were matched by sex (female or male) and age (years). For each case selected, control who is in the same sex and within the same 10-year age group was selected (for example, if the case is aged 27 years, then control was chosen who aged between 20 and 29 years).

### 2.3. Inclusion Criteria

All patients attended the RDTC in the outpatient clinic, inpatient ward, referred to the centre from other departments and consented to the study.

### 2.4. Exclusion Criteria

We excluded patients who have been taking proton pump inhibitors or any antibiotics within four weeks.

### 2.5. Sample Size and Power

The Epi Info computer program version 6 was used for sample size determination. We used 95% confidence interval (CI), a power of 90%, and a proportion of 72% and 40% for *H*. *pylori* among cases and control group respectively, as found in Tanzania and Iran [3, 10]. The parameters provided a total of 55 cases and 55 matched control patients.

### 2.6. Case Selection

For every patient, a detailed history and a thorough physical examination were conducted by the principal investigator (PI) and supervised by the consultant dermatologist. A consecutive sampling method was used in this study. Demographic data such as age, sex, gender, area of residence, and medical history were recorded using the questionnaire after the patient's consent. For those less than 18 years, an assent and a written informed consent were given. Those who were diagnosed to have CU were given sterile stool containers and asked to collect stool of about one gram and hand the sample to the PI.

### 2.7. Control Selection

Patients who had other dermatological conditions during the study period were selected consecutively. Those who consented including patients aged less than 18 years were also asked to collect stool samples. The control group was matched with the cases by age and sex.

### 2.8. Data Collection Method

Face-to-face interview was done using a questionnaire. Stool samples were analyzed immediately in the microbiology lab and then stored at –20°C. *H*. *pylori* antigen test (Tellmefast, Biocan, Canada) was used to test stool samples. The test is a rapid diagnostic method for the detection of *H*. *pylori* antigen in the human stool with a sensitivity of 95% and a specificity of 98% [3]. The findings were recorded in the data extraction sheet.

### 2.9. Statistical Analysis

The findings were summarized into medians with their interquartile range (IQR) for age, and for categorical variables, into frequencies with their respective percentages.

Age was tested for normality and was found to be skewed to the right; thus, the Wilcoxon matched-pair signed-rank test was used to test the association between CU and age. Furthermore, for the categorical variables, the test statistic was based on a Mc Nemar's test or Mantel–Haenszel test stratified by matched pair was used to test the association between CU and age, sex, residency area, occupation, personal or family history of atopy, and *H*. *pylori*. Conditional logistic regression was used to determine the strength of association between CU and *H*. *pylori*. A *p* value of less than 0.05 was considered statistically significant, and all *p* values were two-tailed. Statistical analysis was performed using Stata version 14.1 (Stata Corp LP®, College Station, Texas, USA).

## 3. Results

The total number of participants in this study was 110 patients (55 cases and 55 controls), whereby the median age was 31 (IQR 27–45) among controls versus 34 (IQR: 22–46) years among the cases. Furthermore, over one-third (*n* = 19; 34.6%) of controls were aged from 20 to 29 years, and among cases, the majority (*n* = 18; 32.7%) were in the same age group. Both cases and controls had the same number of each sex, with the majority (*n* = 78; 70.9%) being females. More than half (*n* = 37; 67.3%) of the controls and (*n* = 31; 56.4%) cases reside in urban area (Table 1).

There was no significant association between CU and baseline characteristics of the participants (Table 1).

### 3.1. Distribution of *H*. *pylori* among CU patients

**Figure 1:**
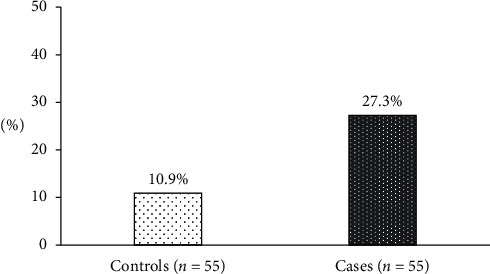
The percentage of the positive test for *H*. *pylori* in matched case and control subjects. The portion was significantly different among cases and controls (*p*=0.0225).

Table 2 shows the matched analysis of the presence of *H*. *pylori* in the case-control pairs in the study. Each member of the pair is either exposed (*H*. *pylori* present) or unexposed (*H*. *pylori* absent) and is a case or a control subject, which yields four possible outcomes. Pairs with the same exposure status for both case and control are concordant pairs, and pairs with different exposures are discordant pairs, such that a total number of concordant pairs = 42 and the total number of discordant pairs = 13. Percentage of subjects who were diagnosed with CU and tested positive for the *H*. *pylori* was significantly different from subjects free from urticaria and tested positive for *H*. *pylori* (15/55 = 27% versus 6/55 = 10.1%; OR: 5.5, 95% CI: 1.2 to 24.8, *p* = 0.0225) (Table 2 and Figure 1).

### 3.2. Crude and Adjusted Conditional Logistic Regression Odds of CU

In the crude analysis, the only variable associated with CU was *H*. *pylori*. For multivariable regression analysis, all the variables which had *p* value ≤0.2 except for age and sex were included in the final model. Backward and forward conditional logistic regression was deployed for multiple regression to determine the predictors of CU. The exposures were adjusted for age and sex of the subjects, area of residence, and the presence of *H*. *pylori* accordingly. The odds of CU among patients who tested positive for *H*. *pylori* were sixfolds higher (OR = 6.2; CI: 1.3–32.3; *p* value 0.029) than those patients who tested negative for *H*. *pylori* (Table 3).

## 4. Discussion

CU is a distressing condition in our everyday practice and is difficult to deal with because of its complex triggering factors [14]. *H*. *pylori* has been implicated as a pathogenic agent in a variety of disorders other than gastrointestinal diseases including CU [15]. This organism can be detected easily by a stool antigen test, which meets the requirements of dermatologists treating most patients with CU because it is rapid, noninvasive, cheap, and convenient for pretreatment diagnosis with a high sensitivity of 95% and a specificity of 98% [9]. The current study aimed to look at the association between CU and *H*. *pylori* infection among patients attending a tertiary hospital in Tanzania.

In this study, a total number of 110 patients were studied (55 cases and 55 controls) with age and gender matching. The majority were aged from 20 to 29 years in both cases and controls, and there was no significant association between CU and baseline characteristics of the participants. Our findings were similar to Muhemmed and Khalis Mohammed, Alfahaad, Moesbehi et al., Majeed, and Jwan Saleh Khoshnaw [3, 4, 9] which explain that CU occurs mostly in adults. Our findings concur with the literature that CU is related to frequent, long duration, and prolonged exposure to allergens or causative agents for immunological reactions to occur [15–17]. These findings were different from the study done by Shin and Lee because their study population was children who aged less than 18 years [18].

We found that 70.9% of the cases and controls were females, while 29.1% of the cases and controls were males. Our findings were similar to Moesbehi et al., and other authors who explained that CU affects more females than males [3, 4, 9, 19]. The reason could be explained by low levels of dehydroepiandrosterone (DHEA)-S in females, suggesting a possible role for hormone imbalance with CU [3, 4]. However, there is limited information from the literature to support this relationship. Muhemmed and Khalis Mohammed suggest that CU is more among females than males because females are more exposed to household activities and household dust. In such activities, they handle raw foods while preparing, hence prone to acquire *H*. *pylori* infection, which will lead to CU more than males [3]. Alfahaad) also explains that females tend to seek medical consultations more compared to males [4].

In this study, 56.4% of the cases and 29.1% of the controls reside in urban areas. Residence area difference may be due to the reason that majority of the patients attending KCMC-RDTC are from inside the town (Moshi). In contrast, those in rural areas usually attend to their health service facilities in their locality.

We found that 27% of CU patients had *H*. *pylori* infection while in controls it was 10.1%. The findings of this study were similar to the studies done in Egypt and Iraq [3, 9, 15], which explain that *H*. *pylori* infection is more in CU patients than in the controls. These similarities could be explained by the fact that Egypt, Iraq, and Tanzania are developing countries, with a higher prevalence of *H*. *pylori* [4, 9, 10]. It was also pointed out by Gu et al., Mogaddam et al., Jaka et al., and Majeed et al. that in these countries, the prevalence of *H*. *pylori* is as high as 90% because there is lack of proper sanitation, basic hygiene, poor diet, and overcrowding that favors *H*. *pylori* transmission, while in developed countries the prevalence is below 40% [8, 10, 15, 17].

In the current study, the only variable which was associated with CU was *H*. *pylori*. We found that the risk of developing CU among patients who were positive for *H*. *pylori* was sixfolds higher compared to those who tested negative, and these findings were statistically significant. These findings were similar to a number of other studies. [3, 9, 15]. It was, however, inconsistent with the findings of Frederman et al. [16]. The reason for this difference could be explained by the different geographical locations, which explains the high prevalence of *H*. *pylori* infection with the risks that favors *H*. *pylori* transmission [3, 4, 17, 20]. In addition, various *H*. *pylori* identification methods are used because most of these studies use the *H*. *pylori* antibody test instead of the antigen test which detects the active infection as used in our study [3, 9, 16].

Several theories have been put forward for this association [3]. It is thought that infection with *H*. *pylori* increases the permeability of the stomach lining and thus increases the exposure to allergens in the gastrointestinal tract [19]. Also, the immune response to *H*. *pylori* produces antibodies that may encourage the release of histamine in the skin [9]. However, in some other studies, this association is controversial as to whether *H*. *pylori* is an etiological agent for CU or not [21, 22].

This association has enormous potential as eradication of the bacteria could signify potential cure [4, 20]. Studies done in the USA, Iran, Pakistan, and Egypt have shown that among patients who had CU, there was complete resolution of the symptoms after eradication of the bacteria [3, 4, 16, 20, 21].

## 5. Conclusion

There is a significant association between CU and *H*. *pylori* infection, indicating to include the *H*. *pylori* tests in the diagnostic workup for CU cases. This organism can be detected with confidence by using rapid, noninvasive, sensitive, specific, and cheaper techniques like serology for the *H*. *pylori* stool antigen test.

## 6. Limitation

This study involved participants who presented at the hospital as dermatology patients; therefore, the results of this study cannot be generalized to the general population.

## 7. Recommendations

We recommend conducting further randomized controlled studies including using *H*. *pylori* eradication drugs.

## Figures and Tables

**Table 1:** Baseline characteristics of case and control subjects after matching.

Characteristics	Control subjects (*n* = 55)	Case subjects (*n* = 55)	*p* value
Median age, years (IQR)	31 (27–45)	34 (22–46)	0.1231^*∗*^

*Age groups (years)*			
<20	9 (16.4)	10 (18.2)	0.3173
20–29	19 (34.6)	18 (32.7)	—
30–39	12 (21.8)	12 (21.8)	—
40–49	8 (14.5)	8 (14.5)	—
>50	7 (12.7)	7 (12.7)	—

*Sex*	—	—	1.0
Male	16 (29.1)	16 (29.1)	—
Female	39 (70.9)	39 (70.9)	—

*Residence area*	—	—	0.2008
Urban	31 (56.4)	37 (67.3)	—
Rural	24 (43.6)	18 (32.7)	—

*Occupational status*	—	—	0.4652
Self-employed	9 (16.4)	13 (23.6)	—
Formal employed	21 (38.2)	17 (30.9)	—
Nonemployed/retired/NA	25 (45.4)	25 (45.5)	—

*Specific attack time*	—	—	—
Cold/hot temp	—	1 (1.8)	—
Not specific	—	54 (98.2)	—

*Associated with angioedema*	—	—	—
Yes	—	10 (18.2)	—
No	—	45 (81.8)	—

*Personal or family history of atopy*	—	—	0.8273
Yes	14 (25.5)	15 (27.3)	—
No	41 (74.5)	40 (72.7)	—

^*∗*^Wilcoxon signed-rank test; NA: not applicable (children).

**Table 2:** Presence of *H*.*pylori* in paired cases and controls (55 matched pairs).

Positive *H*. *pylori* in CU subjects	Positive of *H*. *pylori* in non-CU subjects	
	Yes	No
Yes	4	11
No	2	38

**Table 3:** Odds of association between CU and *H*. *pylori* (*n* = 110).

Variables	COR (95% CI)	*p* values	AOR (95% CI)	*p* value
*Positive H*. *pylori antigen test*^*∗*^				
Yes	5.5 (1.2–24.8)	0.025	6.2 (1.2–32.3)	**0.029**
No	Ref	—	Ref	—

*Sex*				
Male	Ref	—	Ref	—
Female	0.5 (0.4–2.5)	1.0	1.3 (0.4–4)	0.607

Age cantered at the mean (years)	0.9 (0.8–1.1)	0.239	0.9 (0.7–1.1)	0.233

*Area of residence*				
Urban	1.8 (0.7–4.2)	0.207	1.7 (0.6–4.4)	0.287
Rural	Ref	—	Ref	—

*History of treatments* ^*β*^				
Yes	4.5 (0.9–20.8)	0.054	4.8 (0.9–25.1)	0.061
No	Ref	—	Ref	—

^*∗*^Adjusted for age, sex, area of residence, and history of been treated of peptic ulcer/duodenal ulcer/gastritis; ^*β*^been treated of peptic ulcer/duodenal ulcer/gastritis.

## Data Availability

Data are available and will be submitted upon request.

## Abbreviations

CU: Chronic urticaria
*H*. *pylori*: *Helicobacter pylori*
KCMC: Kilimanjaro Christian Medical Centre
MALT: Mucosa-associated lymphoid tissue
RDTC: Regional Dermatology Training Center.
